# Prevalence of Depression and Personality Disorders in the Beginning and End of Emergency Medicine Residency Program; a Prospective Cross Sectional Study

**Published:** 2019-01-25

**Authors:** Farhad Rahmati, Saeed Safari, Behrooz Hashemi, Alireza Baratloo, Roozbeh Khosravi Rad

**Affiliations:** 1 Emergency Medicine Department, Shohadaye Tajrish Hospital, Shahid Beheshti University of Medical Sciences, Tehran, Iran.; 2 Emergency Medicine Department, Sina Hospital, Tehran University of Medical Sciences, Tehran, Iran.

**Keywords:** Depression, Anxiety, Stress, Psychological, Internship and Residency, Emergency Medicine

## Abstract

**Introduction:**

Emergency medicine physicians are constantly under psychological trauma due to encountering critically ill patients, mortality, and violence, which can negatively affect their mental and physical health. The present study was performed with the aim of determining the rate of depression and personality disorders in first-year emergency medicine residents and comparing it with the time they reach the 3rd year.

**Methods:**

In the present prospective cross-sectional study, emergency medicine residents working in multiple teaching hospitals were included via census method and evaluated regarding the rate of depression and personality disorders using the standard MMPI-2 questionnaire upon admission to the program and graduation and their status regarding the evaluated disorders were compared between the 2 phases of evaluation.

**Results:**

99 residents with the mean age of 33.93 ± 5.92 years were evaluated. 85 (85.85%) rated their interest in their discipline as moderate to high. The rates of stress (p = 0.020), anxiety (p < 0.001), and hypomania (p = 0.015) had significantly increased during the 3 years and psychasthenia rate had decreased significantly during this time (p = 0.002). Changes in the prevalence of other disorders on the third year compared to the year of admission to emergency medicine program were not significant.

**Conclusion:**

Considering the results of the present study, it seems that paying more attention to mental problems and decreasing environmental stressors of medical residents, especially emergency medicine residents, should be among the priorities of managers and policymakers of this discipline.

## Introduction:

A considerable part of each individual’s life is spent in the workplace. Environmental factors such as noise, crowding, improper light and sound, human factors like conflict with other individuals, and organizational factors such as work density, improper policy making, injustice and many other factors are among the stressors of workplace. If an individual is not able to effectively cope with these mental pressures, numerous physical, mental, and behavioral side effects will manifest and this will bring about decrease in effectiveness and job dissatisfaction (1).

The rate of anxiety in those working in the field of healthcare is higher than the general population and this is related to long night shifts, low sleeping hours, and high and exhausting workload (2). The emergency department is among the hospital environments with a high tension. Physicians and other emergency staff are constantly under psychological trauma due to encountering critically ill patients, mortality, and violence, which can negatively affect their mental and physical health (3). Emergency physicians experience a high degree of job burnout throughout their career, this rate has been estimated to be about 49% to 65% in emergency medicine residents (4-6). Studies have shown that medical residents experience higher degrees of depression compared to other students (7-11). These facts have received attention from graduate accreditation association and a movement has been initiated for improving physicians’ mental health (12).

Evaluating the health condition of medicine students and evaluating the effects of work environment on their psychological balance seems necessary for better planning and improving the conditions by reducing preventable stressors. Therefore, the present study has been performed with the aim of evaluating the rate of depression and personality disorders in first-year emergency medicine residents and comparing it with the time they reach the 3rd year.

## Methods:

***Study design and setting***

In the present prospective cross-sectional study, all of the first-year emergency medicine residents of Shahid Beheshti University of Medical Sciences, admitted in 2014-2015, were evaluated. The questionnaires used were filled out after obtaining informed consent and by keeping data of the participants completely confidential, once when they entered the program (first year) and once at the time of graduation (third year). The study was approved by the ethics committee of Shahid Beheshti University of Medical Sciences.

***Participants***

Sampling was done using census method and all of the first-year residents, working in teaching hospitals affiliated with the mentioned university, were included without any age or sex limitation. Not giving consent for participation in the study or dropping out of the program and not filling the questionnaire on the third year were exclusion criteria.

***Data gathering***

The tools used for gathering data in this study were baseline characteristics questionnaire and 71-question MMPI2 questionnaire for evaluating the rate of depression and personality disorders. Minnesota Multiphasic Personality Inventory (MMPI) is a standard questionnaire for recalling a broad range of self-described characteristics and scoring them, which gives a quantitative index of the individual’s emotional and their viewpoint on participating in the test (13). All first-year residents filled out the questionnaires in the first phase of the study and their data were recorded. Then 2 years later and in the second phase of the study, the same residents, who had become third-year residents then, filled out the questionnaires again. Finally, the data gathered in the first and third year were compared. In addition, important happenings affecting mental health (such as getting married, having babies, losing dear ones, acute problem in the family, acute disease for the residents themselves,…) that had happened during the 2 years (between the 2 phases of the study) were also recorded to eliminate their confounding effect. The person in charge of data gathering was an emergency medicine resident that personally gathered the data on the first and third year.

***Statistical analysis***

Data were analyzed using SPSS software, version 18. To describe data, mean and standard deviation or frequency and percentage of the variables were used. Before-after test was applied for comparing the condition of personality assessment indices on the first and third year. P<0.05 was considered as level of significance.

## Results:

99 residents with the mean age of 33.93 ± 5.92 (26 – 55) years were evaluated (56.6% female). Table 1 has depicted the baseline characteristics of the studied residents. 85 (85.85%) residents rated their interest in their discipline as moderate to high and only 20 (20.20%) had an income more than 15 million Rials (1500 US dollars) a month. Table 2 has compared the prevalence of depression and other personality disorders at the time the mentioned residents were enrolled in the emergency medicine program with their third year (the time of graduation). Based on the comparison, stress (p = 0.020), anxiety (p < 0.001), and hypomania (p = 0.015) had significantly increased during the 3 years and psychasthenia rate had decreased significantly during this time (p = 0.002). Changes in the prevalence of other disorders on the third year compared to the year of admission to emergency medicine program were not significant.

## Discussion:

Based on the results of the present study, the rate of stress, anxiety and hypomania in the third year emergency medicine residents had significantly increased compared to the time they were first admitted to the residency program and severity of psychasthenia had decreased. Changes in the rate of other disorders on the third year compared to the year of admission to emergency medicine program were not significant. Considering the nature of their job, physicians and healthcare team members are more exposed to stress and anxiety compared to other people in the society (2). Therefore, paying attention to this matter in this group of people is very important. Studies that have been carried out in this regard have reported contradicting results regarding the rate of stress that medical staff members bear; some have reported high rates of stress in surgeons (14) and emergency physicians (15), and in some other studies no significant difference was found regarding stress rate in emergency physicians (16). In another study, results showed that the rate of cortisol measured in residents was not related to the year of residency program they were in (17). However, in another study, the level of stress among professors of medicine had a significant increase after a few years passing (18). This finding shows that increase in cortisol production following stress does not significantly drop with gaining experience (17). An increase in the rate of anxiety following increase in the years of residency in residents was another finding of the present study, which is in line with the study by Buddeberg-Fischer et al. (19).

**Table1:** Baseline characteristics of the studied residents

Variable	**Frequency (%)**
Sex	
Male	43 (43.4)
Female	56 (56.6)
**Age (year)**	
25 – 34.9	59 (59.60)
35 – 44.9	34 (34.34)
> 45	6 (6.06)
**Marital status**	
Married	50 (50.50)
Single	49 (49.50)
**Household breadwinner **	
Yes	29 (29.30)
No	70 (70.70)
**History of consuming tranquilizers**	
Yes	24 (24.24)
No	75 (75.76)
**Smoking**	
Yes	22 (22.22)
No	77 (77.78)
**Family history of psychiatric disorders**	
Yes	23 (23.23)
No	76 (76.77)
**Sports activities**	
Low	49 (49.50)
Moderate	42 (42.42)
High	8 (8.08)
**Interest in emergency medicine**	
Low	14 (14.15)
Moderate	41 (41.41)
High	44 (44.44)
**Monthly outcome (US dollars)**	
500 – 1000	37 (37.38)
1000 – 1500	42 (42.42)
> 1500	20 (20.20)

**Table 2:** Comparing the prevalence of various disorders among the studied residents in their first and third year of emergency medicine residency training

Disorder	**No **	**Mild **	**Moderate **	**severe**	**Very severe**	**P**
**Depression **						
First year	11 (11.1)	34 (34.3)	42 (42.4)	12 (12.1)	0 (0.0)	0.076
Third year	25 (25.3)	27 (27.3)	38 (38.4)	9 (9.1)	0 (0.0)	
**Anxiety **						
First year	29 (29.3)	33 (33.3)	28 (28.3)	6 (6.1)	3 (3.0)	<0.001
Third year	14 (14.1)	19 (19.2)	39 (39.4)	22 (22.2)	5 (5.0)	
**Stress **						
First year	29 (29.3)	28 (28.3)	27 (27.3)	8 (8.1)	7 (7.1)	0.020
Third year	14 (14.1)	21 (21.2)	36 (36.4)	17 (17.2)	11 (11.1)	
**Hypochondriasis**						
First year	6 (6.1)	24 (24.2)	39 (39.4)	28 (28.3)	2 (2.0)	0.074
Third year	3 (3.0)	14 (14.1)	45 (45.5)	28 (28.3)	9 (9.1)	
**Hysteria **						
First year	13 (13.1)	24 (24.2)	37 (37.4)	24 (24.2)	1 (1.0)	0.113
Third year	6 (6.1)	39 (39.4)	29 (29.3)	23 (23.2)	2 (2.0)	
**Psychasthenia**						
First year	25 (25.3)	31 (31.3)	22 (22.2)	13 (13.1)	8 (8.1)	0.002
Third year	24 (24.2)	39 (39.4)	33 (33.3)	3 (3.0)	0 (0.0)	
**Paranoia **						
First year	21 (21.2)	43 (43.4)	16 (16.2)	17 (17.2)	2 (2.0)	0.079
Third year	7 (7.1)	48 (48.5)	20 (20.2)	22 (22.2)	2 (2.0)	
**Schizophrenia **						
First year	27 (27.3)	39 (39.4)	20 (20.2)	13 (13.1)	0 (0.0)	0.316
Third year	18 (18.2)	37 (37.4)	25 (25.3)	19 (19.2)	0 (0.0)	
**Hypomania **						
First year	30 (30.3)	49 (49.5)	18 (18.2)	2 (2.0)	0 (0.0)	0.015
Third year	13 (13.1)	58 (58.6)	21 (21.2)	7 (7.1)	0 (0.0)	

Cabrera et al. (2018) found that emergency medicine residents experience many times more stress and anxiety compared to the general population and this increase in anxiety directly correlates with the increase in their duration of stay and shifts in the emergency department. In this study, no sex difference was observed between the residents regarding anxiety rate (17). 

Overall, it should be noted that in addition to the problems that stress, anxiety, and depression as factors of mental health cause for the resident during education, they also lead to interference with their professional role and taking responsibility of the society’s health in the future. Therefore, it seems that prevention of stress, anxiety and depression of residents and decreasing their mental pressure can play an important role in increasing their interest in working and protecting people’s health in acute and critical situations and cooperating with the group and feeling responsible. Considering the results of the present study, it seems that paying more attention to mental problems of medical residents, especially emergency medicine residents, should be among the priorities of managers and policymakers of this discipline.

***Limitations***

One of the limitations of the present study is its small sample size, which was also seen in previous studies (15, 20). Inability to control some confounding factors such as the menstrual cycle, and personal and family problems were also among the limitations of this study. Another important point is that a control group was not available for performing more comparisons.

## Conclusion:

Based on the results of the present study, the rate of stress, anxiety, and hypomania in the third year emergency medicine residents had significantly increased compared to the time they were first admitted to the residency program and severity of psychasthenia had decreased. Changes in the rate of other disorders on the third year compared to the year of admission to emergency medicine program were not significant.
